# Association between a novel obesity indicator and chronic kidney disease as well as depression: A cross-sectional study based on NHANES 2009 to 2014

**DOI:** 10.1097/MD.0000000000046153

**Published:** 2025-11-28

**Authors:** Wen Du, Kai Zhang, Rui Song

**Affiliations:** a Department of Intensive Care Unit, Xi’an No.3 Hospital, The Affiliated Hospital of Northwest University, Xi’an, Shaanxi, China.

**Keywords:** chronic kidney disease, depression, NHANES, Relative Fat Mass

## Abstract

As a novel, simple, and accessible obesity index, Relative Fat Mass (RFM) provides a more precise estimate of body fat percentage. Current research on the associations of RFM with prevalent chronic kidney disease (CKD) and depression in individuals with CKD remains limited, particularly in representative United States populations. This study included participants from the National Health and Nutrition Examination Survey 2009 to 2014. RFM was calculated using the formula: 64 − (20 × height/waist circumference) + (12 × gender), where gender was coded as 1 (female) and 0 (male). Multivariable-adjusted logistic regression and restricted cubic spline analyses were performed to evaluate the associations of RFM with CKD and with depression in CKD patients. A mediation analysis was conducted to determine whether the association between RFM and CKD was mediated by hypertension and diabetes. Among 12,450 National Health and Nutrition Examination Survey participants, 1285 were diagnosed with CKD. In the fully adjusted logistic regression model, RFM was positively associated with CKD (adjusted odds ratio = 1.02, 95% confidence interval: 1.00–1.04, *P* = .015). Fully adjusted restricted cubic spline analysis showed a linear relationship between RFM and CKD (*P* for non-linear = 0.23), with an optimal cutoff value of 29.6. In addition, hypertension and diabetes mediated 30.34% (*P* < .001) and 21.08% (*P* = .002) of the association between RFM and CKD, respectively. Further fully adjusted logistic regression analysis revealed a positive association between RFM and depression in CKD patients (adjusted odds ratio = 1.05, 95% confidence interval: 1.01–1.08, *P* = .011). In the United States population, elevated RFM is associated with an increased risk of prevalent CKD, and it is also closely linked to a higher risk of depression in individuals with CKD.

## 1. Introduction

Chronic kidney disease (CKD) is a progressive disorder characterized by structural and functional renal alterations from diverse etiologies.^[1]^ Although most patients with CKD remain undiagnosed due to its asymptomatic early course, the estimated prevalence of CKD among United States (U.S.) adults is 15%.^[2,3]^ CKD ranks as a leading cause of death in U.S. adults, with strong associations to multiple comorbidities and adverse outcomes, imposing substantial economic and healthcare burdens.^[4,5]^ Medicare expenditures for CKD in the U.S. exceeded $84 billion in 2017,^[3]^ and recent trends show sustained increases in both incidence and mortality.^[5]^ The pathogenesis of CKD involves complex interactions among metabolic, inflammatory, genetic susceptibility, and environmental/lifestyle determinants.^[6]^ Thus, identifying additional clinical markers with enhanced predictive capacity for CKD onset and progression remains a key priority to mitigate disease incidence and healthcare burden.

Obesity is recognized as a core modifiable risk factor for multiple cardiovascular and metabolic disorders.^[7]^ Over the past 2 decades, accumulating evidence has highlighted a strong association between obesity and CKD. Pathogenic mechanisms linking obesity to kidney injury include activation of the renin–angiotensin–aldosterone system, glomerular hyperfiltration, inflammation, oxidative stress, alterations in leptin/adiponectin balance, dyslipidemia, insulin resistance, and expansion of perirenal and sinus fat-all of which significantly increase the risk of progression to end-stage renal disease.^[7,8]^ While body mass index (BMI) remains the most widely used metric for assessing obesity, it fails to differentiate between adipose tissue and lean body mass.^[9,10]^ The Relative Fat Mass Index (RFM), a novel obesity marker calculated from waist circumference (WC) and height, has been validated by dual-energy X-ray absorptiometry as a more precise indicator of body fat percentage compared to BMI.^[11]^ Although RFM has been linked to various diseases,^[10,12,13]^ its association with CKD in large U.S. population-based cohorts remains undefined.

Additionally, individuals with CKD often experience a high burden of physical symptoms, impaired quality of life, and role dysfunction, which predisposes them to depression.^[14]^ Depression, in turn, further exacerbates poor adherence to medication, fluid, and dietary regimens in CKD patients, contributing to adverse outcomes.^[15–17]^ We further investigated the association between RFM and depression in U.S. adults with CKD. In summary, this study aims to leverage a nationally representative National Health and Nutrition Examination Survey (NHANES) sample to examine the relationship between RFM and the prevalence of CKD in the U.S. population, as well as its association with depression in individuals with CKD.

## 2. Methods

### 2.1. Study design and population

**Figure 1.**
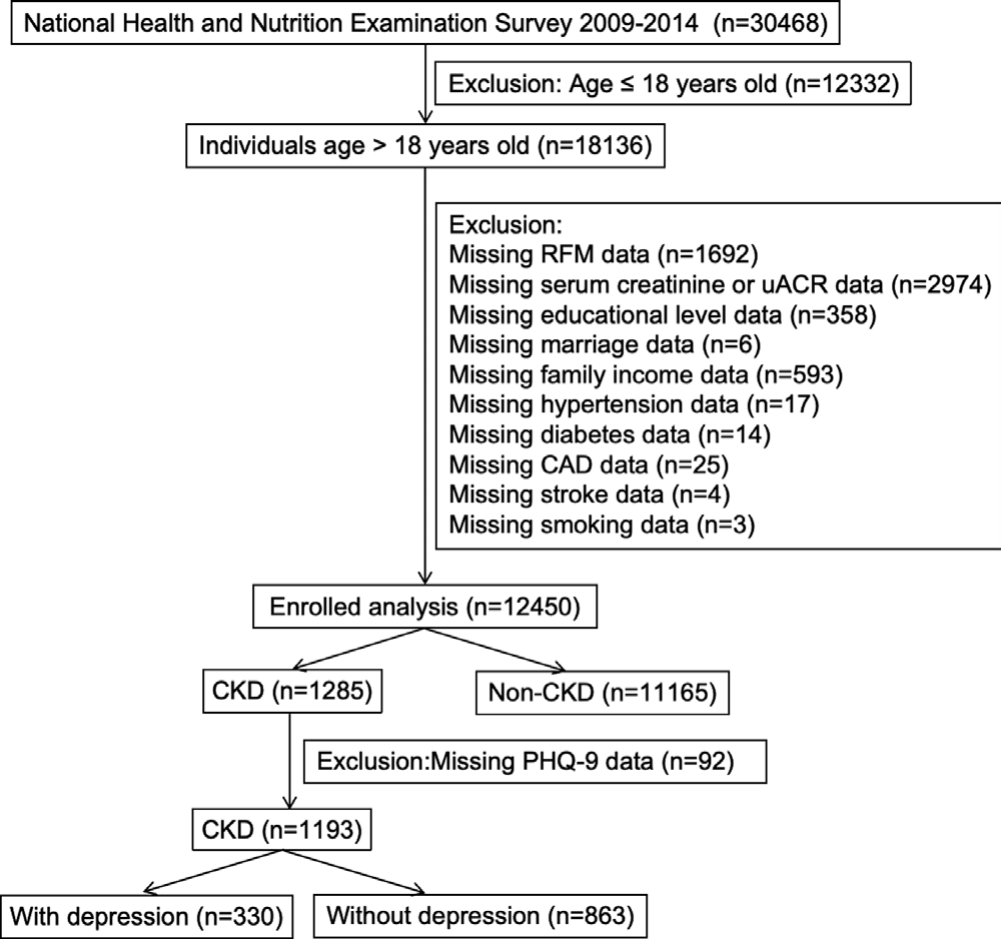
The research flow chart. CAD = coronary artery disease, CKD = chronic kidney disease, PHQ = Patient Health Questionnaire, RFM = Relative Fat Mass, uACR = urine albumin to creatinine ratio.

The Centers for Disease Control administers NHANES, a cross-sectional survey designed to gather information on the health, nutritional status, and health-related behaviors of noninstitutionalized civilian residents in the U.S.^[18]^ Data from NHANES are made publicly available in 2-year intervals, employing a multistage probability sampling approach to ensure representation of the entire U.S. civilian population. This study included 30,468 participants from NHANES 2009 to 2014. We excluded 12,332 individuals aged ≤18 years and 5686 participants with missing data, yielding a final analytic sample of 12,450, including 1285 CKD cases and 11,165 non-CKD controls. In the retained 12,450 participants, all individuals were categorized into 3 tertiles based on specific RFM values: tertile 1 (T1) (RFM ≤ 27.10, n = 4152), T2 (RFM: 27.11–31.88, n = 4150), and T3 (RFM > 31.88, n = 4148). Subsequently, 92 of 1285 CKD patients with missing Patient Health Questionnaire (PHQ-9) data were excluded, leaving 1193 participants (including 330 with and 863 without depression) for assessing the association between RFM and depression (Fig. 1). The NHANES protocol complied with the Health and Human Services Policy for Protection of Human Research Subjects and received approval from the National Center for Health Statistics Ethics Committee. Prior to inclusion in NHANES, all participants provided signed written informed consent. Access to all pertinent data is available at: https://www.cdc.gov/nchs/nhanes/.

### 2.2. Relative Fat Mass

Anthropometric measurements were precisely obtained and recorded by trained NHANES health technicians and recorders in mobile examination centers following standardized protocols, ensuring that measurement discrepancies across NHANES surveys primarily reflect true biological variations. Height was measured using a professional stadiometer with participants barefoot, standing against a vertical board, maintaining a horizontal head position. WC was assessed at the midaxillary line above the iliac crest at the end of normal exhalation, with measurements accurate to within 0.1 cm. The RFM was calculated using the formula: 64 − (20 × height/WC) + (12 × sex), where sex = 0 for males and 1 for females.^[19]^

### 2.3. Diagnosis of CKD and depression

First, the modification of diet in renal disease (MDRD) study equation was used to calculate estimated glomerular filtration rates (eGFRs).^[20]^ In line with contemporary recommendations, we also incorporated the estimation of eGFR using 2 equations: the chronic kidney disease-epidemiology (CKD-EPI) 2009 and the CKD-EPI 2021.^[21]^ According to current guidelines, CKD was defined as an eGFR < 60 mL/min/1.73 m² or a urine albumin-to-creatinine ratio ≥ 30 mg/g, or both.^[22]^

The PHQ-9 is a depression screening tool aligned with DSM-IV criteria for major depressive disorder, consisting of 9 questions about depressive symptoms over the past 2 weeks.^[23]^ Each item is scored 0 to 3, with higher total scores indicating greater depressive severity. Depression has a high prevalence among patients with CKD and is associated with adverse outcomes. Recent studies also have shown that even mild depressive symptoms are closely associated with all-cause mortality in patients with CKD.^[24]^ When the PHQ-9 score is ≥5, the risk of death in CKD patients increases linearly.^[24]^ Therefore, we adopted a PHQ-9 score of ≥5 as the criteria for assessing depression.

### 2.4. Covariates

Demographic information including sex, age, race, marital status, education level, smoking status, and annual household income was obtained via NHANES questionnaires administered by trained professionals. Physical measurements (weight, height, waist circumference) were collected by certified examiners. BMI was calculated as weight (kg) divided by height (m) squared. Diabetes was defined as current use of insulin/oral hypoglycemics or self-reported prior diabetes diagnosis. Hypertension was defined as self-reported hypertension, systolic blood pressure ≥140 mm Hg, diastolic blood pressure ≥90 mm Hg, or use of antihypertensive medications. History of coronary heart disease and stroke was obtained from self-reported questionnaires.

### 2.5. Statistical methods

Given NHANES’s complex, multistage, and stratified probability sampling design, all analyses incorporated sample weights, clustering, and stratification to ensure national representativeness. Therefore, NHANES 2009 to 2014 sampling weights were used to ensure our analyses provide unbiased, accurate performance estimates for the entire population. For continuous variables and categorical variables, baseline data were described as mean ± standard deviation and percentages, respectively.

Multivariable logistic regression models were used to evaluate the associations between RFM (both as a continuous and categorical variable) and CKD, reporting odds ratios (OR) and 95% confidence intervals (CI). Three models were constructed: Model 1 was unadjusted; Model 2 adjusted for sex and age; Model 3, based on Model 2, additionally adjusted for education level, marital status, annual family income, hypertension, diabetes, coronary artery disease, lifetime smoking (≥100 cigarettes), and stroke. Stratified and interaction analyses were then conducted to evaluate whether the association between RFM and CKD varied across different subgroups. Additionally, the magnitudes of associations between RFM, BMI, WC and CKD were compared across 3 logistic models. Receiver operating characteristic (ROC) curves were used to evaluate the discrimination ability of RFM for CKD and depression.

Further, a multivariable-adjusted restricted cubic spline (RCS) analysis with 4 knots set at the 5th, 35th, 65th, and 95th percentiles of the distribution was used to evaluate the nonlinear relationship between RFM and CKD. This analysis also helped determine the optimal RFM cutoff value for CKD prevalence, defined as the point where the OR equals 1 on the RCS curves. Similarly, among CKD-diagnosed participants, 3 logistic models were used to assess the associations of RFM, BMI, and WC with depression: Model 1 unadjusted, Model 2 adjusted for age and sex, Model 3 adjusted for all potential confounders.

In addition, the CKD-2009 and CKD-2021 equations were used to reevaluate CKD to demonstrate the robustness of the results of the correlation between RFM and CKD. Considering the impact of the excluded samples due to data deficiency, we further conducted multiple imputation on the excluded samples using the “mice” R package and carried out a sensitivity analysis to enhance the robustness of the research results. Finally, a mediation analysis was conducted to determine whether the association between RFM and CKD was mediated by hypertension and diabetes. A nonparametric bootstrapping method with 1000 resamples was used to derive CI and assess statistical significance. All data analyses were executed with R version 4.3.2 (R Foundation for Statistical Computing, Vienna, Austria), and the level of significance was set at *P* < .05.

## 3. Results

### 3.1. Baseline characteristics

As shown in Figure 1, a total of 12,450 participants (mean age: 46.34 ± 16.32 years old; 49.21% male) were included in our final analysis and were stratified into 3 tertiles. Baseline characteristics across the 3 RFM groups are presented in Table 1, with all measured variables showing significant differences between groups (all *P* values < .05). The T1 group had the youngest age, the highest proportion of participants with education more than high school, the lowest rates of hypertension, diabetes, coronary artery disease, and stroke. Additionally, the prevalence of CKD was significantly higher in the T3 group than in the T2 and T1 groups (11.96% vs 8.07% vs 4.47%; *P* < .001).

**Table 1:** Baseline characteristics according to RFM tertiles for CKD.

Variables	Overall	T1 (≤27.10)	T2 (27.11–31.88)	T3 (>31.88)	*P*-value
Number	12,450	4152	4150	4148	
RFM index	28.86 (5.48)	23.02 (3.15)	29.50 (1.36)	35.04 (2.46)	<.001
BMI (m/kg^2^)	28.69 (6.50)	23.13 (2.64)	28.16 (2.67)	35.85 (5.90)	<.001
WC (cm)	98.49 (15.83)	83.76 (7.56)	98.50 (6.91)	115.83 (12.13)	<.001
Age (yr)	46.34 (16.32)	40.63 (15.39)	48.55 (15.86)	50.62 (15.96)	<.001
Age group, n (%)					<.001
≤60	9111 (78.39)	3528 (87.98)	2906 (76.14)	2677 (69.57)	
>60	3339 (21.61)	624 (12.02)	1244 (23.86)	1471 (30.43)	
Sex, n (%)					<.001
Male	6125 (49.21)	2236 (51.36)	2274 (54.56)	1615 (40.76)	
Female	6325 (50.79)	1916 (48.64)	1876 (45.44)	2533 (59.24)	
Race, n (%)					<.001
Mexican American	1760 (8.20)	315 (4.97)	702 (9.59)	743 (10.46)	
Other Hispanic	1161 (5.34)	330 (4.88)	411 (5.46)	420 (5.73)	
Non-Hispanic White	5639 (69.00)	1942 (70.04)	1857 (69.83)	1840 (66.87)	
Non-Hispanic Black	2461 (10.29)	803 (9.72)	734 (8.68)	924 (12.76)	
Other	1429 (7.17)	762 (10.39)	446 (6.44)	221 (4.17)	
Educational level, n (%)					<.001
Less than high school	2845 (15.53)	660 (11.33)	1031 (16.61)	1154 (19.28)	
High school	2742 (21.18)	833 (18.40)	90,300 (20.81)	1006 (24.87)	
More than high school	6863 (63.29)	2659 (70.27)	2216 (62.58)	1988 (55.85)	
Marital status, n (%)					<.001
Married/living as married	7510 (64.16)	2341 (59.83)	2757 (70.26)	2412 (62.51)	
Separated/divorced/widowed	2551 (17.35)	650 (13.19)	826 (17.22)	1075 (22.40)	
Never married	2389 (18.49)	1161 (26.97)	567 (12.53)	661 (15.09)	
Smoking ≥ 100 cigarettes in life, n (%)					.12
Yes	5452 (43.48)	1749 (41.82)	1859 (44.14)	1844 (44.70)	
No	6998 (56.52)	2403 (58.18)	2291 (55.86)	2304 (55.30)	
Annual family income, n (%)					<.001
0–14,999 $	1615 (8.58)	472 (7.58)	504 (7.75)	639 (10.67)	
15,000–34,999 $	3959 (24.45)	1136 (21.02)	1327 (23.61)	1496 (29.42)	
–64,999 $	2906 (24.26)	940 (22.56)	962 (23.94)	1004 (26.63)	
≥65,000 $	3970 (42.72)	1604 (48.84)	1357 (44.70)	1009 (33.29)	
Hypertension, n (%)					<.001
Yes	4070 (29.35)	672 (13.86)	1362 (30.36)	2036 (46.50)	
No	8380 (70.65)	3480 (86.14)	2788 (69.64)	2112 (53.50)	
Diabetes, n (%)					<.001
Yes	1484 (9.34)	144 (2.79)	421 (7.62)	919 (18.97)	
No	10,966 (90.66)	4008 (97.21)	3729 (92.38)	3229 (81.03)	
Coronary artery disease, n (%)					<.001
Yes	420 (2.84)	70 (1.33)	156 (3.08)	194 (4.35)	
No	12,030 (97.16)	4082 (98.67)	3994 (96.92)	3954 (95.65)	
Stroke, n (%)					<.001
Yes	357 (2.19)	65 (1.50)	111 (1.78)	181 (3.45)	
No	12,093 (97.81)	4087 (98.50)	4039 (98.22)	3967 (96.55)	
Chronic kidney disease, n (%)					<.001
Yes	1285 (7.96)	223 (4.47)	434 (8.07)	628 (11.96)	
No	11,165 (92.04)	3929 (95.53)	3716 (91.93)	3520 (88.04)	

BMI = body mass index, CKD = chronic kidney disease, RFM = relative fat mass, T = tertile, WC = waist circumference.

Table S1, Supplemental Digital content, https://links.lww.com/MD/Q760 presents baseline comparisons across RFM tertiles among 1193 diagnosed CKD patients, of whom 330 (27.66%) had depression. The T1 group had the younger age and lower prevalences of hypertension, diabetes, coronary artery disease, and stroke compared with T2 and T3 (All *P* values < .05). The T3 group exhibited significantly higher PHQ-9 scores (3.76 ± 4.51 vs 3.11 ± 4.67 vs 2.81 ± 3.45; *P* = .007) than T2 and T1 groups.

### 3.2. Association between RFM and CKD

**Figure 2.**
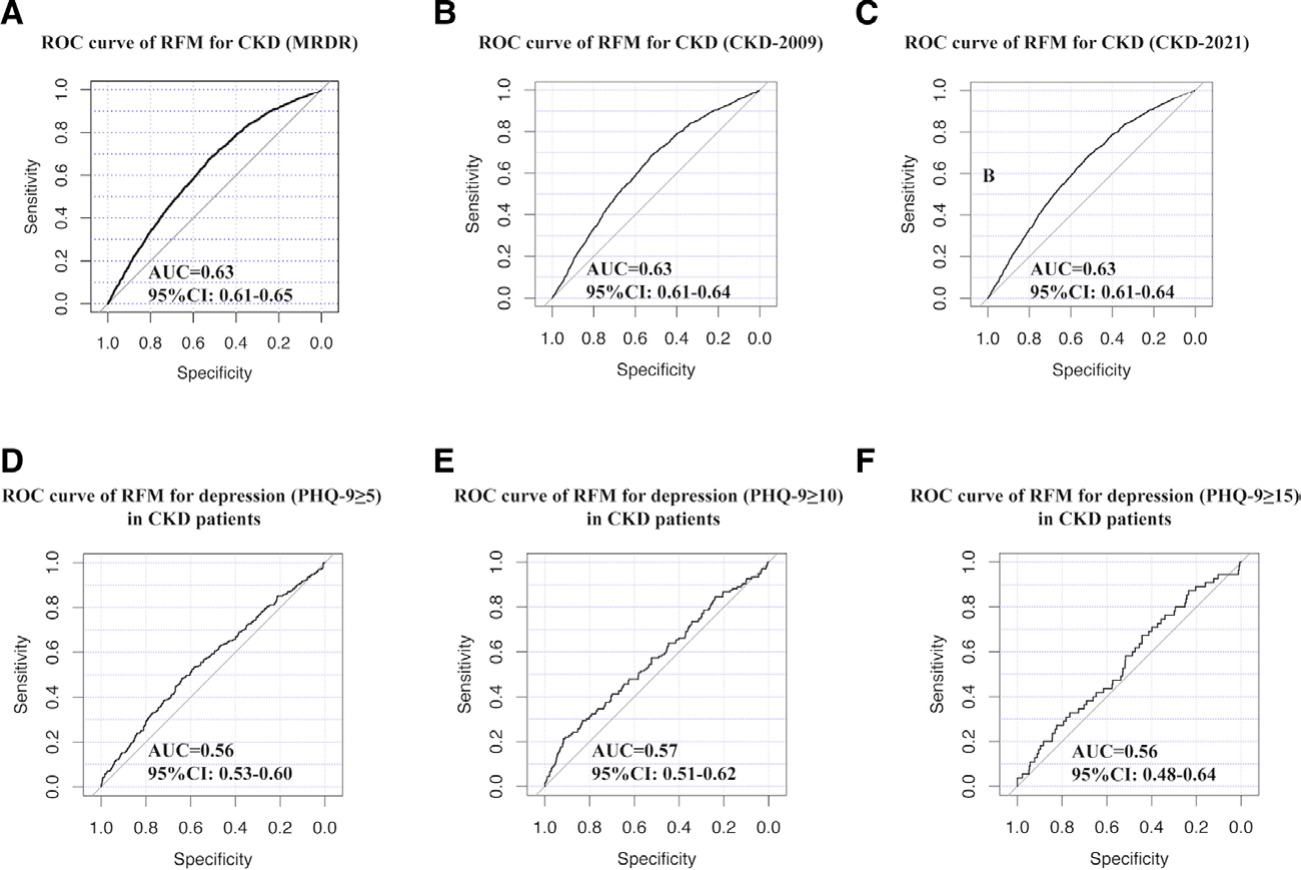
ROC curves indicate that RFM had a certain degree of discrimination ability for CKD and depression in CKD patients. AUC = area under the curve, CI = confidence interval, CKD = chronic kidney disease, CKD-EPI = chronic kidney disease-epidemiology, MDRD = modification of diet in renal disease, PHQ = Patient Health Questionnaire, RFM = Relative Fat Mass, ROC = receiver operating characteristic.

**Figure 3.**
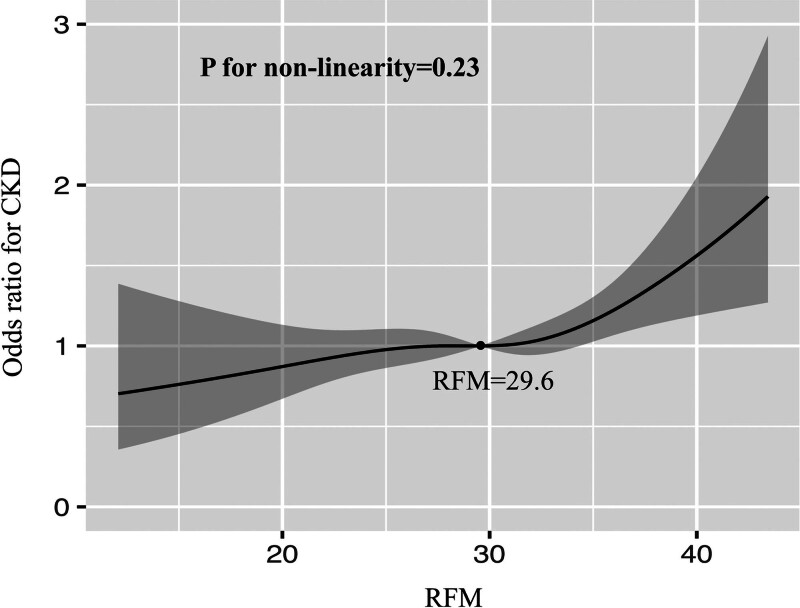
Multivariable-adjusted RCS analysis between RFM and CKD. CKD = chronic kidney disease, RCS = restricted cubic spline, RFM = Relative Fat Mass.

When RFM was treated as a continuous variable, significant positive associations were observed between RFM and CKD in both the unadjusted Model 1 and partially adjusted Model 2. This positive association remained robust in the fully adjusted Model 3 (Table 2; adjusted odds ratio [aOR] = 1.02, 95% CI: 1.00–1.05, *P* = .015). This indicated that each one-unit increase in RFM was associated with a 2% increased risk of CKD. Additionally, in the fully adjusted model 3, each one-unit increase in BMI or WC was associated with a 1% increased risk of prevalent CKD. When RFM was analyzed as a categorical variable, the second and third tertiles both showed a significantly higher risk of prevalent CKD compared with the first tertile in the fully adjusted model (Table 2; T2 vs T1: aOR = 1.36, 95% CI: 1.07–1.73, *P* = .014; T3 vs T1: aOR = 1.33, 95% CI: 1.05–1.69, *P* = .019). ROC curve analysis demonstrated RFM exhibited a certain degree of discriminative ability for prevalent CKD (Fig. 2A; area under the curve [AUC] = 0.63, 95% CI: 0.61–0.65). Finally, multivariable-adjusted RCS analysis revealed a linear relationship between RFM and CKD (Fig. 3; *P* for non-linear = 0.23), with an optimal RFM cutoff value of 29.6.

**Table 2:** Multivariate logistic regression analysis of RFM for CKD.

Exposure	Model 1	Model 2	Model 3
	OR (95% CI), *P*-value	OR (95% CI), *P*-value	OR (95% CI), *P*-value
BMI	1.03 (1.02–1.04), *P* < .001	1.04 (1.02–1.05), *P* < .001	1.01 (1.00–1.03), *P* = .045
WC	1.02 (1.02–1.03), *P* < .001	1.02 (1.01–1.02), *P* < .001	1.01 (1.00–1.01), *P* = .025
RFM (continuous)	1.09 (1.07–1.11), *P* < .001	1.06 (1.04–1.08), *P* < .001	1.02 (1.00–1.04), *P* = .015
*RFM groups*			
T1	Reference	Reference	Reference
T2	2.01 (1.61–2.52), *P* < .001	1.52 (1.20–1.94) *P* = .001	1.36 (1.07–1.73), *P* = .014
T3	3.03 (2.48–3.71), *P* < .001	1.92 (1.53–2.42), *P* < .001	1.33 (1.05–1.69), *P* = .019

Model 1: unadjusted.

Model 2: adjusted for age and sex.

Model 3: adjusted for multivariate variables: age, sex, race, education level, marital status, annual family income, hypertension, diabetes, coronary artery disease, smoking ≥ 100 cigarettes in life, and stroke.

BMI = body mass index, CI = confidence interval, CKD = chronic kidney disease, OR = odds ratio, RFM = Relative Fat Mass, T = tertile, WC = waist circumference.

### 3.3. Association between RFM and depression in CKD patients

A significant positive association was observed between RFM and depression in the fully adjusted Model when RFM was treated as a continuous variable (Table 3; aOR = 1.05, 95% CI: 1.01–1.08, *P* = .011). BMI and WC also showed positive associations with depression in the fully adjusted Model. Each one-unit increase in RFM, BMI, and WC was associated with a 5%, 4%, and 1% increased risk of depression, respectively. When analyzed as a categorical variable, the highest RFM tertile had a significantly higher depression risk compared to the lowest tertile (Table 3; aOR = 1.71, 95% CI: 1.07–2.74, *P* = .025). ROC curve analysis demonstrated a certain predictive value of RFM for depression in CKD patients (Fig. 2D; AUC = 0.56, 95% CI: 0.53–0.60).

**Table 3:** Multivariate logistic regression analysis of RFM for depression in CKD patients.

Exposure	Model 1	Model 2	Model 3
	OR (95% CI), *P*-value	OR (95% CI), *P*-value	OR (95% CI), *P*-value
BMI	1.05 (1.02–1.07), *P* < .001	1.04 (1.02–1.07), *P* < .001	1.04 (1.01–1.06), *P* = .003
WC	1.01 (1.01–1.02), *P* = .001	1.02 (1.01–1.03), *P* < .001	1.01 (1.00–1.02), *P* = .013
RFM (continuous)	1.05 (1.02–1.09), *P* = .003	1.06 (1.02–1.09), *P* = .001	1.05 (1.01–1.08), *P* = .011
*RFM groups*			
T1	Reference	Reference	Reference
T2	1.33 (0.87–2.04), *P* = .18	1.45 (0.91–2.32) *P* = .12	1.46 (0.92–2.32), *P* = .11
T3	1.82 (1.20–2.76), *P* = .006	1.94 (1.23–3.05), *P* = .005	1.71 (1.07–2.74), *P* = .025

Model 1: unadjusted.

Model 2: adjusted for age and sex.

Model 3: adjusted for multivariate variables: age, sex, race, education level, marital status, annual family income, hypertension, diabetes, coronary artery disease, smoking ≥ 100 cigarettes in life, and stroke.

BMI = body mass index, CI = confidence interval, CKD = chronic kidney disease, OR = odds ratio, RFM = Relative Fat Mass, T = tertile, WC = waist circumference.

### 3.4. Subgroup analysis

We further conducted subgroup analyses to investigate the association between RFM tertiles and prevalent CKD stratified by sex, age, smoking status, hypertension, diabetes, coronary artery disease, and stroke. As shown in Table 4. The relationship between RFM and prevalent CKD interacts with age group, sex, hypertension, and coronary artery disease. Specifically, RFM appears to have a stronger association with prevalent CKD among participants who are ≤60 years old, male, non-hypertensive, and without coronary artery disease.

**Table 4:** Subgroup analysis of RFM for CKD.

Subgroup	OR (95% CI)	*P*-value	*P* for interaction
Age (yr)			<.001
≤60	1.09 (1.07, 1.11)	<.001	
>60	1.04 (1.02, 1.06)	<.001	
Gender			.003
Male	1.11 (1.09, 1.13)	<.001	
Female	1.07 (1.06, 1.09)	<.001	
Smoking ≥ 100 cigarettes in life			.83
Yes	1.09 (1.07, 1.10)	<.001	
No	1.09 (1.07, 1.11)	<.001	
Hypertension			.002
Yes	1.03 (1.01, 1.04)	.001	
No	1.07 (1.05, 1.09)	<.001	
Diabetes			.45
Yes	1.07 (1.05, 1.08)	<.001	
No	1.06 (1.03, 1.09)	<.001	
Coronary artery disease			.036
Yes	1.04 (0.99, 1.08)	.12	
No	1.09 (1.07, 1.10)	<.001	
Stroke			.061
Yes	1.04 (0.99, 1.09)	.089	
No	1.09 (1.07, 1.10)	<.001	

*Note*: adjusted for age, sex, race, educational level, marital status, smoking, annual family income, hypertension, diabetes, coronary artery disease, and stroke except the subgroup variable.

Calculate the OR and CI by taking RFM tertiles as continuous variables.

CI = confidence interval, CKD = chronic kidney disease, OR = odds ratio, RFM = Relative Fat Mass.

### 3.5. Sensitivity analysis

Additionally, the CKD-EPI 2009 and CKD-EPI 2021 equations were used to calculate eGFR for reevaluating CKD. Further analyses were conducted to explore the associations of RFM with prevalent CKD and with depression in patients with CKD. The results were consistent with the primary analysis: higher RFM consistently exhibited a stronger positive association with prevalent CKD (Table S2, Supplemental Digital content, https://links.lww.com/MD/Q760; CKD-EPI 2009: T3 vs T1, aOR = 1.52, 95% CI: 1.16–1.99, *P* = .003; CKD-EPI 2021: T3 vs T1, aOR = 1.52, 95% CI: 1.20–1.93, *P* = .001) and with depression in CKD patients (Table S2, Supplemental Digital content, https://links.lww.com/MD/Q760; CKD-EPI 2009: T3 vs T1, aOR = 1.68, 95% CI: 1.07–2.64, *P* = .025; CKD-EPI 2021: T3 vs T1, aOR = 1.96, 95% CI: 1.24–3.10, *P* = .005). Moreover, ROC curve analysis showed that RFM had comparable discriminative ability for prevalent CKD, regardless of whether eGFR was calculated using the MDRD, CKD-EPI 2009, or CKD-EPI 2021 equation (Fig. 2A for MDRD; AUC = 0.63, 95% CI: 0.61–0.65; Fig. 2B for CKD-EPI 2009; AUC = 0.63, 95% CI: 0.61–0.64; Fig. 2C for CKD-EPI 2021; AUC = 0.63, 95% CI: 0.61–0.64). Additionally, RFM demonstrated a certain degree of discriminative value for depression in CKD patients, where depression was assessed using PHQ-9 scores with cutoffs of 5 and 10 (Fig. 2D; AUC = 0.56, 95% CI: 0.53–0.60; Fig. 2E; AUC = 0.57, 95% CI: 0.51–0.62). However, RFM showed poor discriminative ability for depression in CKD patients when depression was assessed using a PHQ-9 score cutoff of 15 (Fig. 2F; AUC = 0.56, 95% CI: 0.48–0.64).

Finally, to address potential bias introduced by excluding samples with missing data, multiple imputation was performed on the data. As shown in Table S3, Supplemental Digital content, https://links.lww.com/MD/Q760, in the analysis of the imputed data, each one-unit increase in RFM was associated with a 3% increased risk (aOR = 1.03, 95% CI: 1.01–1.05, *P* < .001) of prevalent CKD in the fully adjusted model. The second and third tertiles both showed a significantly higher risk of prevalent CKD compared with the first tertile (Table S3, Supplemental Digital content, https://links.lww.com/MD/Q760; T2 vs T1: aOR = 1.37, 95% CI: 1.10–1.70, *P* = .007; T3 vs T1: aOR = 1.52, 95% CI: 1.21–1.90, *P* = .004).

### 3.6. Mediation analysis

**Figure 4.**
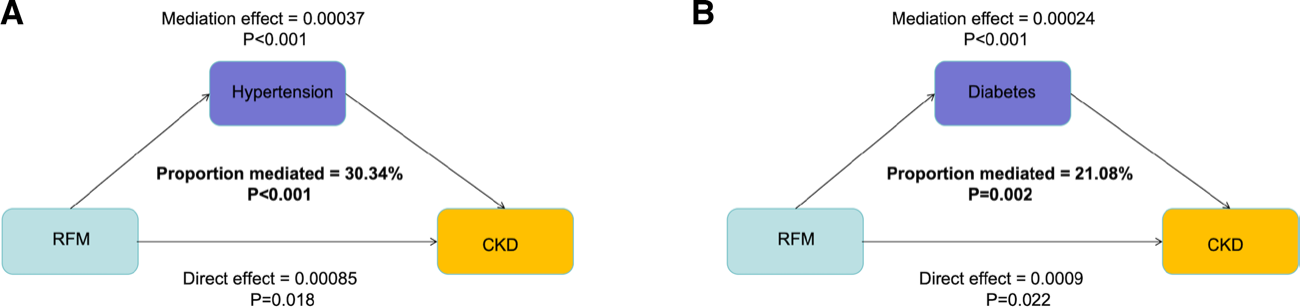
Estimated proportion of the association between RFM and CKD mediated by hypertension and diabetes. CKD = chronic kidney disease, RFM = Relative Fat Mass.

To evaluate whether hypertension and diabetes exert a potential mediating effect on the relationship between RFM and prevalent CKD, a mediation analysis was performed. The results showed that in the fully adjusted model, the mediating effects of hypertension and diabetes on the association between RFM and prevalent CKD were 0.00037 (*P* < .001) and 0.00024 (*P* < .001), respectively, with corresponding mediation proportions of 30.34% and 21.08% (Fig. 4A and B).

## 4. Discussion

This large-sample cross-sectional study investigated the associations of RFM with CKD and with depression among CKD patients. We found that higher RFM levels were associated with increased CKD risk, with each unit increase in RFM conferring a relatively greater risk than BMI or WC. A linear relationship was observed between RFM and prevalent CKD. RFM was positively associated with risk of depression among CKD patients. Additionally, hypertension and diabetes mediated 30.34% and 21.08% of the association between RFM and CKD, respectively (Graphical Abstract).

Obesity and CKD are 2 conditions with high prevalence that impose substantial global economic and healthcare burdens.^[25]^ The “cardio-renal-metabolic syndrome” proposed by the American Heart Association has been defined as a disorder associated with the interplay of obesity, diabetes, CKD, and cardiovascular diseases, highlighting the importance of metabolism and adiposity for vital organs like the heart and kidneys.^[26]^ The association between obesity and CKD has been widely corroborated in previous studies. For example, the Framingham Offspring Study cohort enrolled participants without preexisting kidney disease and found that higher BMI was associated with a greater risk of developing CKD after a mean follow-up of 18.5 years.^[27]^ Another large-scale study in multiethnic populations identified a significant linear correlation between BMI and the risk of end-stage renal disease.^[28]^ Additionally, Mendelian randomization studies have demonstrated a causal relationship between obesity and CKD.^[29]^ Obesity exerts adverse effects on renal function through direct and indirect pathways. Direct effects include obesity-related glomerulopathy and renal hemodynamic changes. Indirect mechanisms may involve activation of the renin–angiotensin–aldosterone system, hypertension, adipokines leading to renal lipotoxicity, and the nucleotide-binding oligomerization domain-like receptor pyrin domain 3 inflammasome, all of which can impact renal function.^[25]^

Previous investigations into obesity have predominantly relied on BMI, yet this metric has inherent limitations: it cannot distinguish between muscle and adipose tissue, nor can it differentiate central obesity from general adiposity.^[9,30]^ Recent studies have shown that central obesity exhibits a stronger association with diseases than general obesity. Developed by U.S. researchers, the RFM is a novel obesity index that provides a more accurate estimate of total body fat percentage compared with BMI.^[11]^ Additionally, large cohort studies in the U.S., Brazil, and South Korea have demonstrated that RFM correlates more strongly with obesity measured by dual-energy X-ray absorptiometry than does BMI.^[13]^ In recent years, RFM has been shown to have stronger associations with the prognosis of various diseases than BMI.^[31–33]^ However, no study to date has clarified the association between RFM and CKD in a representative large U.S. population, nor whether RFM-a simple measurable index-exhibits a stronger association with CKD than BMI and WC. A comparative study of the U.S. and China found rising prevalence of CKD in both nations, but a declining mortality trend in China versus an increasing trend in the U.S. This underscores the importance of identifying simple markers with strong CKD associations in the U.S. population.^[5]^ In this general U.S. sample, RCS analysis determined the optimal RFM cutoff for the prevalence of CKD as 29.6, though requiring confirmation through interventional studies.

Additionally, the prevalence of depression in individuals with CKD is exceptionally high, and depression can reduce medication and fluid management adherence in this population, increasing the incidence of adverse outcomes.^[14]^ Previous studies have shown a close association between obesity and depression. Our study further found that after adjusting for confounders, RFM was more strongly associated with depression than BMI and WC in individuals with CKD. This research has several strengths. First, it utilized large nationally representative data in the U.S., enhancing the reliability of the findings. Second, RFM is a simple metric that can even be self-measured at home, and unlike dual-energy X-ray, it involves no radiation or additional costs. However, several limitations should not be overlooked. First, as a cross-sectional study, it cannot establish a causal relationship between RFM and CKD or depression. Second, due to sample limitations, it is impossible to further conduct stratified sensitivity analysis by eGFR/urine albumin-to-creatinine ratio category and CKD stage. Third, the study is based on a U.S. sample, so caution is warranted when generalizing conclusions to other regions. Since our current findings based cross-sectional NHANES do not establish a causal relationship, further prospective studies are needed to confirm the associations of RFM with CKD and with depression in CKD patients. Interventional studies targeting RFM are also required to determine whether RFM modification confers beneficial outcomes.

## 5. Conclusion

In conclusion, RFM was positively associated with prevalent CKD in U.S. adults, with an optimal cutoff value of 29.6. Additionally, RFM was also positively associated with depression in U.S. patients with CKD.

## Acknowledgments

To all who participated in NHANES and lent a hand, our heartfelt gratitude.

## Author contributions

**Conceptualization:** Wen Du, Rui Song.

**Data curation:** Wen Du, Kai Zhang.

**Formal analysis:** Wen Du, Kai Zhang.

**Investigation:** Rui Song.

**Methodology:** Wen Du.

**Project administration:** Rui Song.

**Software:** Wen Du, Kai Zhang.

**Supervision:** Rui Song.

**Validation:** Rui Song.

**Writing – original draft:** Wen Du, Kai Zhang.

**Writing – review & editing:** Rui Song.

## Supplementary Material


