# Supplementary material for: Association between a novel obesity indicator and chronic kidney disease as well as depression: A cross-sectional study based on NHANES 2009 to 2014
Source: Medicine (Baltimore). 2025 Nov 28;104(48):e46153. doi: 10.1097/MD.0000000000046153 (PMC12662556; doi:10.1097/MD.0000000000046153)
Supplement: Supplementary file 1 [file medi-104-e46153-s001.docx]

| **Table S1. Baseline characteristics according to RFM tertiles for depression in CKD patients** | | | | | |
| --- | --- | --- | --- | --- | --- |
| Variables | Overall | T1 (≤ 27.10) | T2 (27.11-31.88) | T3 (> 31.88) | P-value |
| RFM index | 31.10 (5.13) | 23.63 (3.18) | 29.58 (1.37) | 35.44 (2.50) | <0.001 |
| BMI (m/kg^2^) | 30.16 (7.23) | 22.43 (2.62) | 27.09 (2.67) | 35.74 (6.40) | <0.001 |
| WC (cm) | 103.61 (16.68) | 83.17 (7.80) | 97.81 (7.07) | 116.67 (12.76) | <0.001 |
| Age (years) | 63.46 (14.43) | 59.89 (16.87) | 63.53 (14.66) | 64.95 (12.78) | 0.011 |
| Age group, n (%) |  |  |  |  | 0.03 |
| ≤ 60 | 388.00 (36.74) | 85.00 (45.72) | 116.00 (39.87) | 187.00 (30.58) |  |
| > 60 | 805.00 (63.26) | 120.00 (54.28) | 276.00 (60.13) | 409.00 (69.42) |  |
| Sex, n (%) |  |  |  |  | 0.039 |
| Male | 547.00 (41.72) | 100.00 (38.28) | 207.00 (49.30) | 240.00 (37.70) |  |
| Female | 646.00 (58.28) | 105.00 (61.72) | 185.00 (50.70) | 356.00 (62.30) |  |
| Race, n (%) |  |  |  |  | 0.036 |
| Mexican American | 121.00 (4.62) | 7.00 (1.80) | 37.00 (4.44) | 77.00 (5.97) |  |
| Other Hispanic | 80.00 (3.48) | 9.00 (2.68) | 29.00 (3.48) | 42.00 (3.82) |  |
| Non-Hispanic White | 603.00 (73.38) | 105.00 (74.27) | 195.00 (73.75) | 303.00 (72.71) |  |
| Non-Hispanic Black | 333.00 (14.77) | 65.00 (14.35) | 112.00 (14.73) | 156.00 (14.98) |  |
| Other | 56.00 (3.76) | 19.00 (6.90) | 19.00 (3.60) | 18.00 (2.51) |  |
| Educational level, n (%) |  |  |  |  | 0.043 |
| Less than high school | 334.00 (19.85) | 51.00 (18.50) | 96.00 (16.84) | 187.00 (22.62) |  |
| High school | 303.00 (24.58) | 53.00 (22.37) | 101.00 (23.91) | 149.00 (26.03) |  |
| More than high school | 556.00 (55.57) | 101.00 (59.12) | 195.00 (59.24) | 260.00 (51.36) |  |
| Marital status, n (%) |  |  |  |  | <0.001 |
| Married/living as married | 657.00 (60.13) | 117.00 (63.97) | 232.00 (63.33) | 308.00 (56.15) |  |
| Separated/divorced/widowed | 430.00 (31.69) | 59.00 (19.96) | 134.00 (31.73) | 237.00 (36.74) |  |
| Never married | 106.00 (8.18) | 29.00 (16.07) | 26.00 (4.94) | 51.00 (7.11) |  |
| Smoking≥100 cigarettes in life, n (%) |  |  |  |  | 0.72 |
| Yes | 612.00 (48.94) | 107.00 (47.04) | 206.00 (51.10) | 299.00 (48.20) |  |
| No | 581.00 (51.06) | 98.00 (52.96) | 186.00 (48.90) | 297.00 (51.80) |  |
| Annual family income, n (%) |  |  |  |  | 0.23 |
| 0-14999 $ | 209.00 (12.16) | 28.00 (9.66) | 69.00 (11.33) | 112.00 (13.84) |  |
| 15000-34999 $ | 418.00 (30.39) | 74.00 (27.07) | 130.00 (27.82) | 214.00 (33.69) |  |
| 35000-64999 $ | 298.00 (26.91) | 58.00 (32.80) | 96.00 (25.71) | 144.00 (25.22) |  |
| ≥65000 $ | 268.00 (30.55) | 45.00 (30.47) | 97.00 (35.14) | 126.00 (27.25) |  |
| Hypertension, n (%) |  |  |  |  | <0.001 |
| No | 393.00 (37.57) | 97.00 (54.93) | 144.00 (43.10) | 152.00 (26.03) |  |
| Yes | 800.00 (62.43) | 108.00 (45.07) | 248.00 (56.90) | 444.00 (73.97) |  |
| Diabetes, n (%) |  |  |  |  | <0.001 |
| No | 840.00 (74.15) | 182.00 (90.28) | 302.00 (81.79) | 356.00 (61.61) |  |
| Yes | 353.00 (25.85) | 23.00 (9.72) | 90.00 (18.21) | 240.00 (38.39) |  |
| Coronary artery disease, n (%) |  |  |  |  | 0.66 |
| Yes | 130.00 (10.91) | 21.00 (11.47) | 45.00 (9.31) | 64.00 (11.83) |  |
| No | 1,063.00 (89.09) | 184.00 (88.53) | 347.00 (90.69) | 532.00 (88.17) |  |
| Stroke, n (%) |  |  |  |  | 0.11 |
| Yes | 103.00 (8.01) | 14.00 (8.99) | 32.00 (5.14) | 57.00 (9.67) |  |
| No | 1,090.00 (91.99) | 191.00 (91.01) | 360.00 (94.86) | 539.00 (90.33) |  |
| PHQ-9 score | 3.35 (4.39) | 2.81 (3.45) | 3.11 (4.67) | 3.76 (4.51) | 0.007 |
| Abbreviations: RFM, relative fat mass; CKD, chronic kidney disease; BMI: body mass index; WC, waist circumference; T, tertile. | | | | | |

| **Table S2. Sensitivity analysis for CKD assessment using the CKD-EPI 2009 and CKD-EPI 2021 equations** | | | | |
| --- | --- | --- | --- | --- |
|  | **CKD-EPI 2009 (OR, 95% CI, P-value)** | | **CKD-EPI 2021 (OR, 95% CI, P-value)** | |
| Exposure | RFM for CKD | RFM for Depression in CKD patients | RFM for CKD | RFM for Depression in CKD patients |
| BMI | 1.02 (1.01-1.03), P=0.018 | 1.04 (1.01-1.06), P=0.008 | 1.02 (1.01-1.03), P=0.005 | 1.04 (1.01-1.06), P=0.003 |
| WC | 1.01 (1.00-1.01), P=0.012 | 1.01 (1.00-1.02), P=0.012 | 1.01 (1.00-1.02), P=0.002 | 1.01 (1.00-1.02), P=0.007 |
| RFM (continuous) | 1.03 (1.01-1.05), P=0.007 | 1.05 (1.00-1.08), P=0.038 | 1.03 (1.01-1.05), P=0.002 | 1.05 (1.01-1.08), P=0.018 |
| RFM groups |  |  |  |  |
| T1 | Reference | Reference | Reference | Reference |
| T2 | 1.26 (0.95-1.65), P=0.10 | 1.23 (0.70-2.16) P=0.46 | 1.26 (1.01-1.58), P=0.044 | 1.06 (0.65-1.72), P=0.80 |
| T3 | 1.52 (1.16-1.99), P=0.003 | 1.68 (1.07-2.64), P=0.025 | 1.52 (1.20-1.93), P=0.001 | 1.96 (1.24-3.10), P=0.005 |
| Logistic model adjusted for multivariate variables including age, sex, race, education level, marital status, annual family income, hypertension, diabetes, coronary artery disease, smoking≥100 cigarettes in life, and stroke; Abbreviations: RFM, relative fat mass; CKD, chronic kidney disease; CKD-EPI, chronic kidney disease-epidemiology; BMI, body mass index; WC, waist circumference; T, tertile; OR, odds ratio; CI, confidence interval. | | | | |
|  |  |  |  |  |
|  |  |  |  |  |
|  |  |  |  |  |
|  |  |  |  |  |

| **Table S3. Multivariate logistic regression analysis of RFM for CKD after multiple imputation** | | | |
| --- | --- | --- | --- |
| Exposure | Model 1 OR (95% CI), P-value | Model 2  OR (95% CI), P-value | Model 3 OR (95% CI), P-value |
| BMI | 1.00 (1.00-1.00), P<0.001 | 1.00 (1.00-1.00), P<0.001 | 1.00 (1.00-1.00), P=0.038 |
| WC | 1.00 (1.00-1.00), P<0.001 | 1.00 (1.00-1.00), P<0.001 | 1.00 (1.00-1.00), P=0.006 |
| RFM (continuous) | 1.09 (1.07-1.10), P<0.001 | 1.08 (1.07-1.10), P<0.001 | 1.03 (1.01-1.05), P<0.001 |
| RFM groups |  |  |  |
| T1 | Reference | Reference | Reference |
| T2 | 2.02 (1.66-2.47), P<0.001 | 1.80 (1.46-2.21), P<0.001 | 1.37 (1.10-1.70), P=0.007 |
| T3 | 3.15 (2.62-3.78), P<0.001 | 2.83 (2.34-3.43), P<0.001 | 1.52 (1.21-1.90), P=0.004 |
| Model 1: unadjusted;  Model 2: adjusted for age and sex; Model 3: adjusted for multivariate variables: age, sex, race, education level, marital status, annual family income, hypertension, diabetes, coronary artery disease, smoking≥100 cigarettes in life, and stroke; Abbreviations: RFM, relative fat mass; CKD, chronic kidney disease; BMI: body mass index; WC, waist circumference; T, tertile; OR, odds ratio; CI, confidence interval. | | | |
|  |  |  |  |
|  |  |  |  |
|  |  |  |  |
|  |  |  |  |
